# Reciprocal Associations Between Normative, Affectual, and Associational Solidarity With Parents in Young Adults

**DOI:** 10.1093/geroni/igaa057.1652

**Published:** 2020-12-16

**Authors:** Jeung Hyun Kim, Woosang Hwang, Kent Jason Cheng, Maria Brown, Merril Silverstein

**Affiliations:** Syracuse University, Syracuse, New York, United States

## Abstract

Intergenerational solidarity has become important as close family ties mobilize the provision of social support across generations and contribute to the family wellbeing. One popular approach to studying intergenerational cohesion in aging families is through the theoretical construct of intergenerational solidarity. However, less is known about the longitudinal and reciprocal associations between normative, affectual, and associational solidarity with mothers and fathers among young-adult children in the transition to adulthood. On the basis of the theoretical construct of intergenerational solidarity, we examined the reciprocal associations between three dimensions of intergenerational solidarity (normative, affectual, and associational) with parents in young-adult children from their early twenties to late thirties. Data were derived from 287 mother-son, 325 mother-daughter, 262 father-son, and 297 father-daughter groups who participated in the Longitudinal Study of Generations between 2000 and 2016. Autoregressive cross-lagged model with latent variables predicted the causal relations between three dimensions of solidarity across four parent-child groups. We found that young-adult sons’ perceived associational solidarity with parents predicted normative solidarity over time, whereas young-adult daughters’ perceived affectual solidarity with mothers predicted normative solidarity over time. In addition, young-adult daughters’ perceived normative solidarity predicted affectual solidarity for fathers over time. The present study found that young-adult sons and daughters have different ways establishing normative solidarity in their early twenties to late thirties according to parents’ gender. In addition, this study found that normative solidarity is beneficial for young-adult daughters developing emotional closeness with fathers over time.

